# Mega-scale single-cell profiling reveals novel biomarkers associated with acute GvHD after allogeneic hematopoietic stem cell transplantation

**DOI:** 10.1186/s40364-025-00868-x

**Published:** 2025-12-01

**Authors:** Zheng Song, Evgeny Klyuchnikov, Anita Badbaran, Likai Tan, Regine J. Dress, Emilia Czajkowski, Simeon Weßler, Radwan Massoud, Christine Wolschke, Anja Schimrock, Yu Zhang, Cedric Ly, Nico Gagelmann, Kristin Rathje, Boris Fehse, Stefan Bonn, Sarina Ravens, Nicola Gagliani, Christian F. Krebs, Ulf Panzer, Francis Ayuk, Nicolaus Kröger, Immo Prinz

**Affiliations:** 1 Institute of Systems Immunology, University Medical Center Hamburg-Eppendorf, Martinistr. 52, 20251 Hamburg, Germany; 2 Hamburg Center for Translational Immunology, University Medical Center Hamburg-Eppendorf, Martinistr. 52, 20251 Hamburg, Germany; 3 Department of Stem Cell Transplantation, University Medical Center Hamburg-Eppendorf, Martinistr. 52, 20251 Hamburg, Germany; 4 Department of Anaesthesia and Intensive Care and Peter Hung Pain Research Institute, The Chinese University of Hong Kong, 4/F Main Clinical Block and Trauma Centre, Prince of Wales Hospital, Shatin, New Territories, Hong Kong SAR, China; 5 Li Ka Shing Institute of Health Sciences, The Chinese University of Hong Kong; 4/F Main Clinical Block and Trauma Centre, Prince of Wales Hospital, Shatin, New Territories, Hong Kong SAR, China; 6 Institute of Immunology, Hannover Medical School; Carl-Neuberg-Str. 1, 30625 Hannover, Germany; 7 Institute of Medical Systems Bioinformatics, Center for Biomedical AI (bAIome), Center for Molecular Neurobiology (ZMNH), University Medical Center Hamburg-Eppendorf, Falkenried 94, 20251 Hamburg, Germany; 8 Department of General, Visceral and Thoracic Surgery, University Medical Center Hamburg-Eppendorf, Martinistr. 52, 20251 Hamburg, Germany; 9 I. Department of Medicine, University Medical Center Hamburg-Eppendorf, Martinistr. 52, 20251 Hamburg, Germany; 10 III. Department of Medicine, University Medical Center Hamburg-Eppendorf, Martinistr. 52, 20251 Hamburg, Germany; 11 Hamburg Center for Kidney Health (HCKH), University Medical Center Hamburg-Eppendorf, Martinistr. 52, 20251 Hamburg, Germany

**Keywords:** Mega-scale scRnAseq, AML, alloHSCT, αβ and γδ T cell persistence, aGvHD, GvL

## Abstract

**Background:**

Alloreactive T cells mediate graft-versus-leukemia (GvL) reactions and acute graft-versus-host disease (aGvHD) in AML patients following allogeneic hematopoietic stem cell transplantation.

**Methods:**

To investigate biomarkers that identify alloreactive T cells associated with either beneficial GvL or detrimental aGvHD, we collected graft samples and two post-transplant follow-up blood samples (day 30 and day 100) of ten AML patients undergoing hematopoietic stem cell transplantation and profiled over 777,000 CD45^+^ leukocytes in total by combinatorial barcoding-based mega-scale single-cell RNA sequencing.

**Results:**

Using immune receptor sequences as intrinsic clonal barcodes, we observed that especially CD8^+^ graft-derived T cells persisted and displayed enhanced proliferation, clonal expansion, and likely alloreactivity. Notably, patient-derived peripheral leukocytes that survived the conditioning, as identified by sex-chromosome-related genes, were primarily CD4^+^ T helper cells. MDGA1 expression on T cells and NK cells emerged as a novel biomarker potentially associated with aGvHD. Additionally, we observed a significant deficiency of ADGRG1 expression, a marker of alloreactive cytotoxic T cells, by αβ and γδ T cells from relapsed patients.

**Conclusions:**

In conclusion, mega-scale single-cell monitoring of graft and hematopoietic immune cell reconstitution allowed us to demonstrate that MDGA1 and ADGRG1 may function as complementary biomarkers expressed by distinct circulating T cells that are associated with divergent outcomes in AML patients, enabling precise risk stratification of alloHSCT outcomes and presenting potential therapeutic targets.

**Graphical Abstract:**

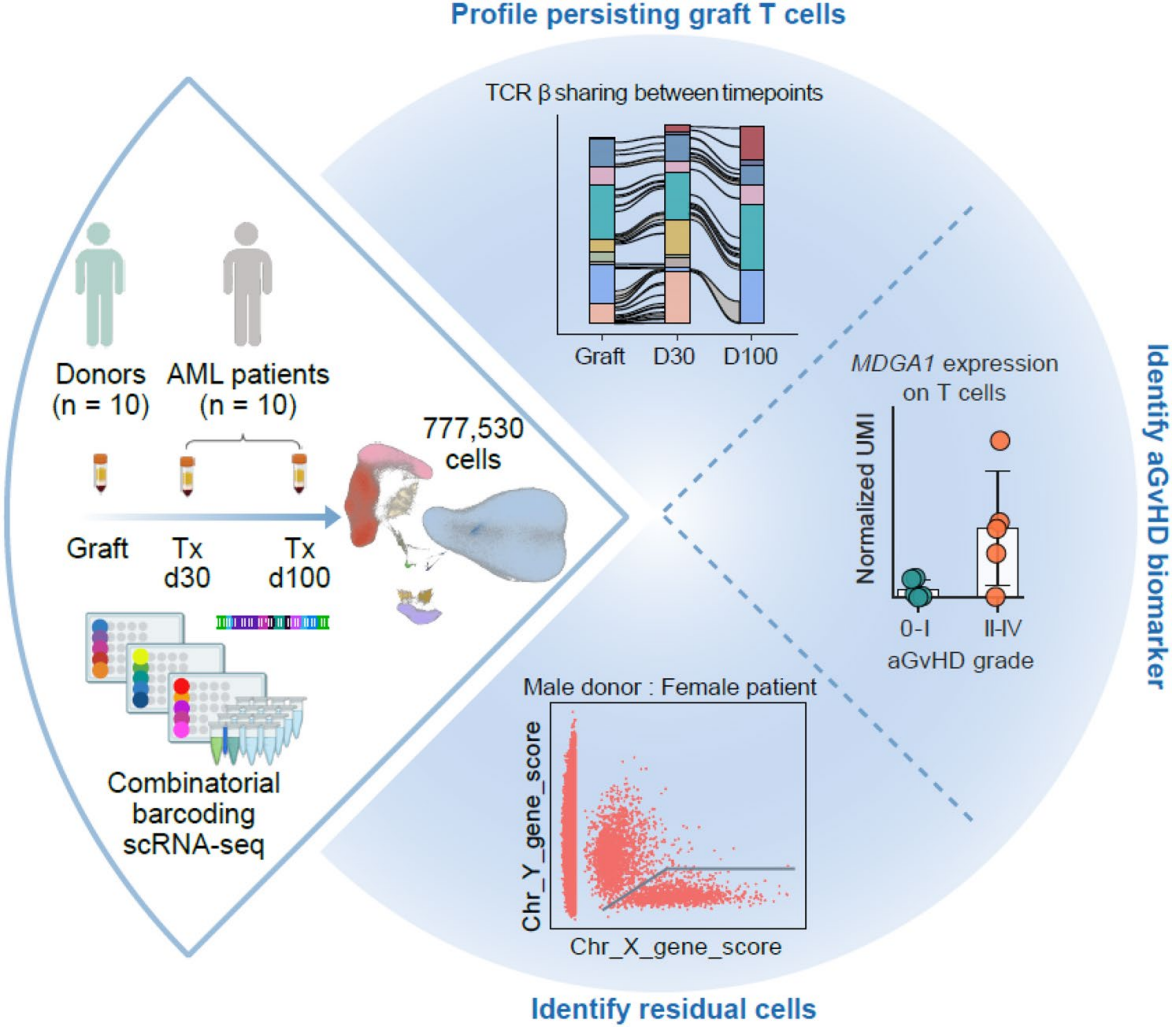

**Supplementary Information:**

The online version contains supplementary material available at 10.1186/s40364-025-00868-x.

## Background

Allogeneic hematopoietic stem cell transplantation (alloHSCT) remains the only curative therapy in acute myeloid leukemia (AML). [1] In alloHSCT, the elimination of residual leukemia cells surviving the preparative regimen is primarily driven by engrafted alloreactive lymphocytes, especially by αβ T cells through the GvL effect. [2, 3] However, alloreactive T cells responding to alloantigens and/or minor histocompatibility antigens on recipient cells are also involved in the pathogenesis of graft-versus-host disease (GvHD). [4, 5] Based on clinical symptoms, GvHD is classified as acute GvHD (aGvHD) or chronic GvHD, with aGvHD characterized by severe inflammation typically affecting the gastrointestinal tract, skin, and liver[6–8].

The failure of graft-versus-leukemia (GvL) reactivity leads to relapse (REL) which is the main cause of post-alloHSCT mortality. [9] To overcome this, clinical strategies aim at augmenting GvL while minimizing GvHD through graft composition modification. [10] However, there is a positive correlation between GvL and GvHD in AML patients. [11–13] Notably, depleting alloreactive αβ T cells from graft before infusion has shown significant improvement of the chronic GvHD-free, relapse free survival probability in pediatric leukemia patients. [14, 15] This benefit is partly attributed to natural killer (NK) cells and γδ T cells, which exert potent antileukemic effects in a major histocompatibility complex (MHC)-independent way [16–18].

Despite advancements in graft engineering, some AML patients undergoing alloHSCT treatment still relapse or develop aGvHD, highlighting the need for identifying robust biomarkers for rapid diagnosis and early interventions of alloHSCT-associated GvL and aGvHD. Recently, using single-cell RNA sequencing (scRNA-seq), *ADGRG1* (GPR56) and *ZNF683* (HOBIT) were identified as biomarkers of alloreactive CD8^+^ cytotoxic T lymphocytes linked to effective antileukemia response, that were significantly enriched in the bone marrow of patients achieving complete remission (CR) following alloHSCT. [19–21] However, whether the predictive value of these biomarkers extends to expression by other cytotoxic T cells, such as γδ T cells, in peripheral blood remains unexplored.

Furthermore, a recent CyTOF-based study revealed that CD11b^+^ CD163^+^ monocytes and CXCR3^+^ T cells appear in the blood and accumulate in the skin and gastrointestinal (GI) tract at the onset of aGvHD [22, 23], highlighting the power of high-throughput technologies in advancing biomarker discovery. Recent advances in scRNA-seq now enable large-scale, transcriptome-wide identification of novel aGvHD biomarkers across all immune cell populations in peripheral blood and biopsy samples [19–21, 24].

## Methods

### Clinical cohort and patient samples

Ten adult ( > 18y) patients with AML in complete remission who underwent alloHSCT with peripheral blood stem cell grafts (matched unrelated, *n* = 8; matched related, *n* = 2) in the Department for Stem Cell Transplantation of University Cancer Center Hamburg-Eppendorf from December 2021 to May 2022 were included in this study (Table 1). The study was approved by ethics commission of the Medical Chamber of Hamburg and was performed according to the approved study protocol (version 1.1 from 01.11.2021). Written informed consent was obtained from all participants. The majority of patients received myeloablative conditioning (*n* = 7) with ATG-based immunoablation (*n* = 9). After a median follow-up of 20 months (18–22), three patients died (relapse, *n* = 2; NRM, *n* = 1). The relapses occurred on days +149 and +622. Five patients developed acute GvHD (II-IV) at a median of 57 days (19–136). The eight patients remained alive in complete remission at the last follow-up.

**Table 1:** A. Clinical characteristics of patients. MUD: Match unrelated donor; MRD: Match related donor; MAC: Myeloablative conditioning; RIC: Reduced-intensity conditioning; BU: Busulfan; Flu: Fludarabine; pCy: Post-transplant cyclophosphamide; TT: Thiotepa; ATG: Anti-thymocyte globulin; Treo: Treosulfan; TBI: Total body irradiation; NRM: Non-relapse mortality. B. Sample information. Available samples for the single-cell experiment are indicated

Patient	Age/sex	ELN 2022	Donor	Conditioning	Protocol	aGvHD (II-IV)	cGvHD	Relapse	NRM	Death
**A**										
REL_1	36/f	intermediate	MUD	MAC	Bu/Flu/pCy	no	0	yes	no	yes
REL_2	68/m	adverse	MUD	RIC	TT/Bu/ATG	yes	0	yes	no	no
CR_1	47/f	intermediate	MUD	MAC	TT/Bu/ATG	yes	moderate	no	no	no
CR_2	56/m	intermediate	MUD	RIC	TT/Bu/ATG	yes	severe	no	no	no
CR_3	38/m	favorable	MRD	MAC	TT/Bu/ATG	no	0	no	no	no
CR_4	52/f	intermediate	MUD	RIC	Treo/Flu/ATG	yes	0	no	yes	yes
CR_5	67/f	intermediate	MUD	MAC	TT/Bu/ATG	yes	0	no	yes	yes
CR_6	68/m	favorable	MUD	MAC	TBI/Flu/ATG	no	moderate	no	no	no
CR_7	68/f	intermediate	MUD	MAC	TBI/Flu/ATG	no	0	no	no	no
CR_8	30/f	favorable	MRD	MAC	TT/Bu/ATG	no	0	no	no	no
**B**										
Patient		Graft sample			D30 sample			D100 sample		
REL_1		no			yes			yes		
REL_2		yes			yes			yes		
CR_1		yes			yes			yes		
CR_2		yes			yes			yes		
CR_3		yes			yes			yes		
CR_4		yes			yes			no		
CR_5		yes			yes			no		
CR_6		yes			yes			no		
CR_7		yes			yes			yes		
CR_8		yes			yes			no		

Peripheral blood stem cell graft samples were collected at the time of transplantation and were cryopreserved on the next day. The PBMC samples of recipients were collected at day +30 (D30) and day +100 (D100) and were cryopreserved on the same or the following day. Mononuclear cells were isolated using a Ficoll gradient and cryopreserved in DMSO until they were used for single cell analysis.

### scRNA-seq library preparation and sequencing

All DAPI^−^ (Thermo Fisher Scientific Cat# D1306) (RRID:AB_2629482) live cells were sorted from frozen PBMC samples at the flow cytometry core facility of University Medical Center Hamburg-Eppendorf (RRID:SCR_017156). The sorted cells were fixed and permeabilized using Evercode™ Cell Fixation v2 (Parse Biosciences, Cat. ECF2001) following the manufacturer’s protocol. Fixed cells were stored at −80 °C. All samples were then processed together to prepare single-cell libraries using one Evercode™ WT Mega v2 Kit (Parse Biosciences, Cat. ECW02050), according to the manufacturer’s instructions. Fifteen sub-libraries were sequenced on the Illumina NovaSeq 6000 S4 flow cell platform (RRID:SCR_016387) using PE150 chemistry with a sequencing depth of 25,000 reads per cell.

### Preprocessing and dimensional reduction of scRNA-seq data

Raw fastq data were aligned to human genome assembly GRCh38 (hg38, Annotation release 109) and quantified using split-pipe software suite (Parse Biosciences, v1.1.1). The filtered feature-barcode matrices containing only cellular barcodes (Valid cell barcodes: 777 920; Median transcripts/cell: 5 931; Median genes/cell: 2 511) were used for further analysis. Single cell gene expression matrices were analyzed using Seurat (5.1.0, RRID:SCR_016341) [25]. To maximize cell retention, a relative broad threshold was first set to clean the data with n_genes < 8 500, n_transcripts < 60 000, and percent_mito < 25%. After normalization, we sketched 10 000 cells of each donor and performed integration on the sketched cells using Harmony algorithm [26] based on donors. Dimensional reduction of the integrated sketched matrix was performed using Uniform Manifold Approximation and Projection (UMAP, RRID:SCR_018217) [27] with the first 14 principal components. Clustering was done by constructing a k-nearest neighbors graph and identifying groups of cells using the original Louvain algorithm [28] with resolution of 2.0. Clusters were annotated based on the expression of canonical marker genes and three populations representing doublets (doublets number: 26,111, doublets ratio: 3.36%) were annotated.

Major immune cell populations—including T cells, NK cells, B cells, monocytes, and dendritic cells—were subset and analyzed using Scanpy (v1.10.3, RRID:SCR_018139) [29, 30]. For each subset, preprocessing was performed using stringent quality control thresholds: n_genes < 5 000, n_transcripts < 15 000, and percent_mito < 10%. Putative doublets were identified and removed using the Scrublet algorithm (RRID:SCR_018098) [31]. For each sample, the top 3000 highly variable genes (HVGs) were identified. Hemoglobin genes, unannotated genes, mitochondrial genes, heat shock proteins, and ribosomal genes were excluded from HVGs. Integration was performed based on samples using Harmony algorithm (RRID:SCR_022206) [26]. Clustering was performed by constructing a k-nearest neighbors graph (k = 10) using the first 40 principal components from Harmony-corrected embeddings. Cell clusters were identified using the Leiden algorithm [32] with a resolution of 0.6. A PAGA [33] graph was computed to infer connectivity between clusters, and UMAP [27] embedding was initialized using the PAGA positions for better preservation of global topology. Clusters were annotated based on the expression of canonical marker genes and by referencing previously published gene modules [34].

γδ T cells were distinguished from αβ T cells based on their transcriptomic profiles using an in-house developed algorithm [35] and were annotated using reference gene modules [36].

### Immune repertoire reconstitution and analysis

Immune repertoire was reconstituted from transcriptome using TRUST4 algorithm (RRID:SCR_026162) [37]. Briefly, TRUST4 performed de novo assembly of V, J, and C gene segments, including the hypervariable complementarity-determining region 3 (CDR3), to generate consensus contigs of BCR/TCR sequences. These contigs were then realigned to IMGT reference gene sequences to annotate V(D)J gene usage and CDR3 features. Only functional BCR/TCR with valid cell barcodes were retained for downstream analysis. CDR3 amino acid sequences were used to define unique clonotypes. Top three clonally expanded TCR β chain were employed to perform clone-matched analysis.

### Differentially expressed genes and enrichment analysis

To mitigate the ‘drop-out’ effect associated with lowly expressed genes in single-cell data, functional analyses of CD8^+^ T cells were performed at the pseudo-bulk level rather than at the single-cell level. Specifically, CD8^+^ T cells from each sample (graft and follow-up) were aggregated by summing gene-level counts to create pseudo-bulk expression profiles. Differential expression analysis (DEA) was conducted using DESeq2 (RRID:SCR_015687) [38], and functional enrichment was assessed using the Over Representation Analysis (ORA) method implemented in decoupleR (RRID:SCR_027127) [39]. Gene set activity was inferred using the Hallmark gene sets from the Molecular Signatures Database (MSigDB) (RRID:SCR_016863) [40].

### Donor-recipient cell demultiplexing based on sex-specific gene expression

To distinguish recipient-derived cells in sex-mismatched post-transplant samples, demultiplexing was performed based on the expression of sex-specific genes. Expression of Y chromosome–linked genes (*UTY*, *DDX3Y*, *USP9Y*, *RPS4Y1*, *RPS4Y2*, *EIF1AY*, *KDM5D*, *PRY*, *PRY2*, *DAZ1*, *DAZ2*, *DAZ4*, *ZFY*, *AMELY*, *TBL1Y*, *PCDH11Y*) and X chromosome–linked genes (*XIST*, *TSIX*) was used to compute gene expression scores (Chr_Y_gene_score and Chr_X_gene_score) using the scanpy.tl.score_genes function from the Scanpy package. Female patient-derived (recipient) cells were identified based on low Chr_Y_gene_score and high Chr_X_gene_score.

### Quantitative PCR to measure MDGA1 expression in T cells

Total RNA was extracted from sorted T cells obtained from three patients without aGvHD and four patients with aGvHD before treatment using the RNeasy Mini Kit (Qiagen, Cat. 74,106) according to the manufacturer’s instructions. RNA concentration and purity were assessed using a Qubit RNA BR Assay Kit (Invitrogen, Cat. Q10210). Complementary DNA (cDNA) was synthesized from 30 ng of total RNA using the SMARTScribe Reverse Transcriptase (Takara, Cat. 639,536). Quantitative PCR (qPCR) was performed on a QuantStudio 3 Real-Time PCR System (Applied Biosystems) using PowerUp SYBR Green PCR Master Mix (Thermo Fisher Scientific, Cat. A25742). Gene-specific primers were designed to amplify MDGA1 and the housekeeping gene GAPDH as an internal control. Each reaction was run in triplicate in a total volume of 10 µL under standard cycling conditions: 95 °C for 10 min, followed by 40 cycles of 95 °C for 15 s and 60 °C for 1 min. Ct values were obtained using the QuantStudio software (v1.7). Relative MDGA1 expression was calculated using the 2^−^ ΔΔCt method, with GAPDH serving as the reference gene and the non-aGvHD group as the reference group. The sequences of qPCR primers are MDGA1_F: CATCCTGACCGATCTCCGTG, MDGA1_R: GTAGTGGATGATGCGGGAGG, GAPDH_F: TCAAGGCTGAGAACGGGAAG, and GAPDH_R: CGCCCCACTTGATTTTGGAG.

### Reanalysis of public datasets

For MS [41] dataset, processed AnnData objects were obtained from the original publications.

For the cytokine treated PBMC dataset [42], we obtained the AnnData object of all PBMCs and subset T cells. After log-normalization, the proportion of *MDGA1*^+^ cells was calculated for each sample. Fold change was computed relative to the PBS-treated baseline within each donor, and log₂ fold change (log₂FC) values were transformed accordingly. Paired statistical comparisons between cytokine-treated and PBS-treated samples from the same donor were performed using two-tailed paired t-tests. To visualize the results, volcano plots were generated by plotting log₂FC against –log₁₀(p-value). Points were color-coded based on statistical significance (*p* < 0.05) and direction of change.

For the alloHSCT transplantation [43] dataset, raw matrix was obtained from the original publications and processed with the code from the authors. *ADGRG1* feature was calculated based on the top 20 upregulated genes of ADGRG1^+^ T cells (*ADGRG1*, *GZMB*, *FGFBP2*, *CX3CR1*, *GZMH*, *EFHD2*, *GNLY*, *NKG7*, *SPON2*, *CLIC3*, *PRF1*, *FCGR3A*, *ZEB2*, *KLRD1*, *TRGC2*, *S1PR5*, *CST7*, *SSBP3*, *TTC38*, *PLEK*) [21] using the *scanpy.tl.score_genes* function from the Scanpy package.

### Statistical analysis

Statistical analyses were performed using using Python (v3.9.18) with the scipy package (v1.12.0) for independent and paired t-tests (ttest_ind, ttest_rel), and Mann-Whitney U test (mannwhitneyu). In Supplementary figures S3–S4, paired t-tests were conducted between selected timepoints (e.g., Graft vs. D30, D30 vs. D100, Graft vs. D100) for donors with matched samples. To control for multiple testing, Bonferroni correction was applied to adjust p-values. In Fig. 3F-G and Fig. 5A-C, two-tailed paired Student’s t-tests were performed to calculate p-values. In Fig. 4A-C, Fig. 4E-F, Fig. 4 H-I, Fig. 6F-H, two-tailed Student’s t-tests were performed to calculate p-values.

## Results

### Longitudinal single-cell immune profiling of AML patients undergoing alloHSCT

**Fig. 1:**
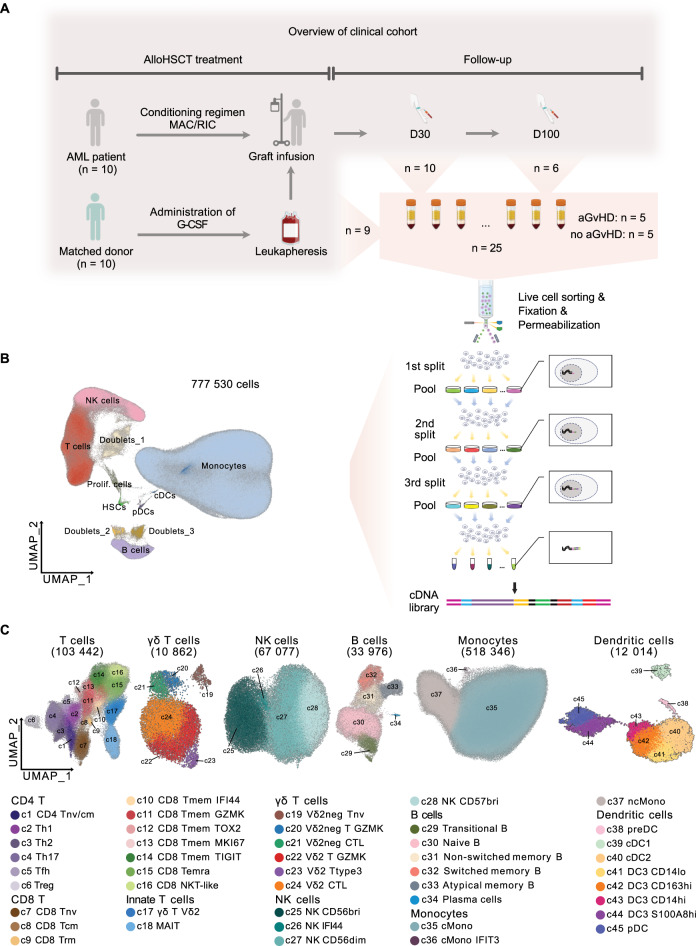
Experimental design and annotation of immune cells. (**A**) Schematic experimental workflow. The number of patients and samples for each timepoint are denoted. AML, acute myeloid leukemia; MAC, myeloablative conditioning; RIC, reduced intensity conditioning; aGvHD, acute graft-versus-host-disease; G-CSF, granulocytes colony stimulating factor. (**B**) Uniform Manifold Approximation and Projection (umap) of 777,530 PBMC transcriptomes (including doublets). Each dot represents a cell or a doublet and is colored based on the cell type. Prolif. cells, proliferating cells; HSCs, hemopoietic stem cells. (**C**) UMAP of T cells, γδ T cells, NK cells, B cells, monocytes, and dendritic cells (DCs) with post-QC cell number indicated. Cells are colored by subtypes and the cluster ID are shown

To profile the immune reconstitution dynamics in AML patients post-alloHSCT, we collected 25 samples from ten patients at three timepoints: prior to graft infusion (Graft, *n* = 9), 30 days post-transplantation (D30, PBMC, *n* = 10), and 100 days post-transplantation (D100, PBMC, *n* = 6) (Table 1). During the observation period, two patients experienced relapse and five developed aGvHD (Table 1). In total, approximately ten million live immune cells were sorted and used to generate scRNA-seq libraries in a single experiment to minimize potential batch effects (Fig. 1A). After quality control, over 770,000 cells from the 25 samples were classified into eight major hematopoietic cell populations and three doublet clusters, with a median of 29,000 cells per sample (Fig. 1B; Fig. S1A-D). Following an additional round of fine-tuned quality control and doublet removal, 45 immune subtypes of αβ T cells, γδ T cells, NK cells, B cells, monocytes, and dendritic cells (DCs) were annotated based on their canonical marker genes (Fig. 1C; Fig. S2A-F).

We next examined the hematopoietic reconstitution patterns after alloHSCT. Within the lymphocyte compartment, quantitative analysis showed a gradual increase of the T cell proportions and a rapid rise in NK cell proportions especially CD56 bright populations at D30, while B cells nearly disappeared at D30 and were only slowly reconstituted at D100 (Fig. S3A, S4A-G). Monocytes accounted for a substantial proportion of the graft samples and show a trend to decrease post-alloHSCT (Fig. S3B, S4H). The overall DC proportion did not change except for an increase of preDC from D30 to D100 and a decrease of cDC1 on D30 (Fig. S3B, S4I-J). Interestingly, the hematopoietic stem cell (HSC) compartment and a population characterized by expression of *MKI67* remained constant at all timepoints (Fig. S3C-D). These findings provide a comprehensive, carefully annotated high resolution landscape of graft and immune reconstitution in AML patients following alloHSCT treatment.

### Graft-derived αβ T cells and γδ T cells persist in the host environment with activated transcriptional profiles consistent with alloreactivity

**Fig. 2:**
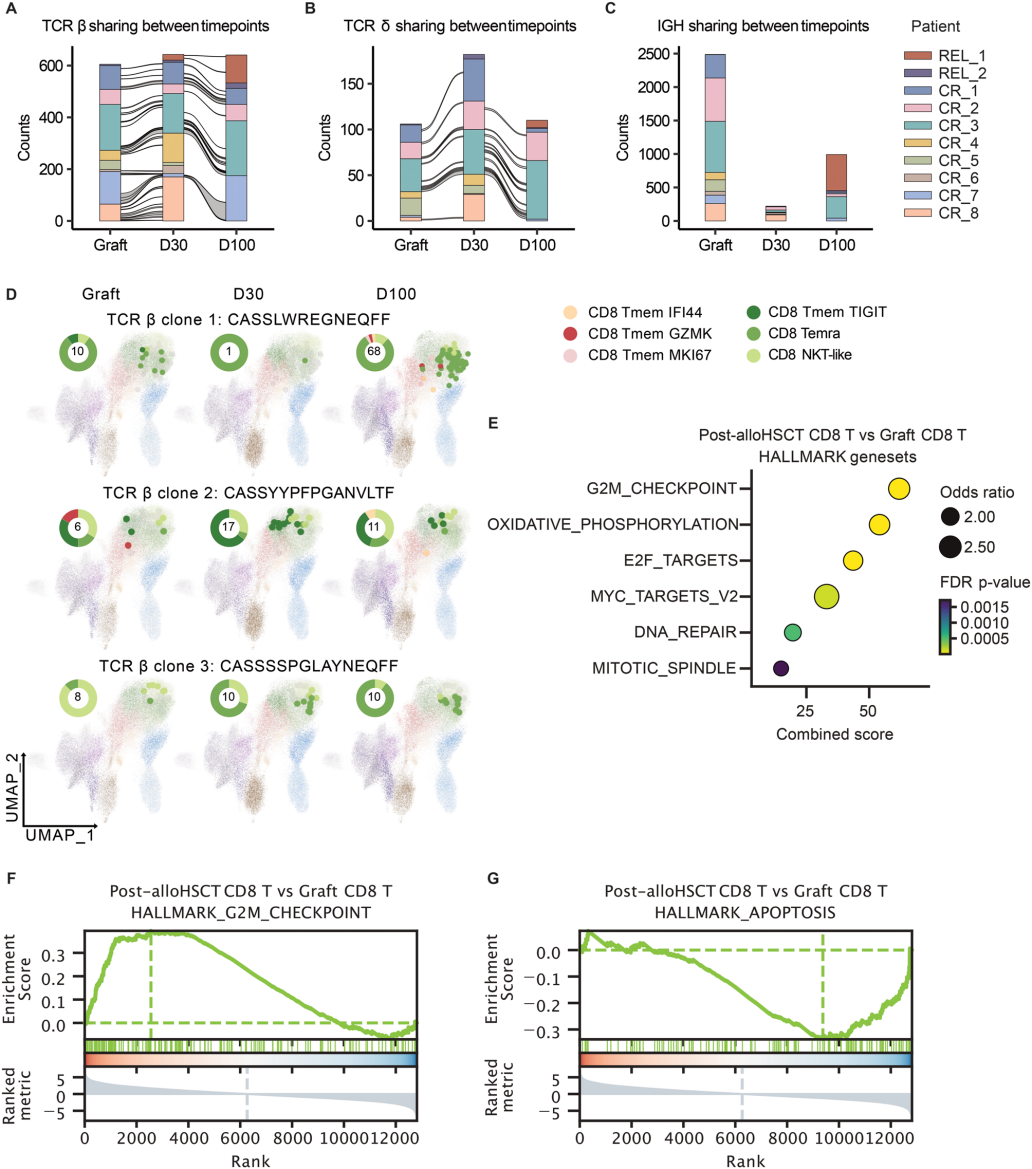
Comparison of immune repertoire overlap and differentially expressed genes of CD8^+^ T cells between graft and follow-up samples. (**A-C**) the Complementary Determined Region 3 sequences (CDR3s) of T Cell Receptor (TCR) beta chain (**A**), TCR delta chain (**B**), and **B** Cell Receptor (BCR) heavy chain (**C**) are used to show the overlap of immune repertoire between different timepoints. (**D**) the three hyperexpanded TCR beta clones are shown on the T cell umap at three timepoints. Cells are colored by T cell subtypes. Pie plots on the shoulder are colored by the subtypes of T cells with indicated clones. The number shows the total T cell number with indicated clones at indicated timepoint.(**E**) Pathway analysis of differentially expressed genes of CD8^+^ T cells. (**F-G**) Enrichment of cell cycle and apoptosis gene signatures on CD8^+^ T cells

The sustained presence of engrafted immune cells in peripheral blood is a key indicator of successful hematopoietic chimerism. To track the graft-derived immune cells over time, the highly variable sequences of immune receptor genes were used as intrinsic molecular identifiers for individual T and B cell clones. We employed the TRUST4 algorithm [37] to reconstruct the αβ/γδ T cell and B cell receptor (TCR/BCR) repertoires from the transcriptomic data (Fig. S5A-C). The CDR3s of TCR β chain, TCR δ chain, and BCR heavy chain, which were distinct among individuals, were used to trace the graft-derived αβ T cells, γδ T cells, and B cells respectively (Fig. S5D-G). We consistently identified multiple shared TCR β and TCR δ clones, but no overlapping immunoglobulin heavy chain (IGH) clones in paired graft and post-alloHSCT samples from the same patients (Fig. 2A–C). These findings indicate that donor-derived αβ and γδ T cell clones persisted in the host circulation after transplantation. At the same time, no persistent donor B cell clones were detected, which is consistent with previous studies. [43–45] The presence of shared TCR clones between graft and post-transplant time points confirmed successful long-term survival of donor T cells.

The most prominent persisting engrafted T cell clones were CD8^+^ T cells. To further characterize their phenotype and differentiation in the host environment, we monitored their clonal expansion and differential gene expression post-alloHSCT. Clone-matched analysis of the top three most expanded TCR β clones revealed expansion and phenotypic conversion of CD8^+^ T cell clones to proliferating CD8^+^ memory or terminally differentiated Temra T cells after alloHSCT (Fig. 2D; Fig. S5H-J). Concurrently, genes upregulated in post-alloHSCT CD8^+^ T cells were significantly enriched in cell cycle–related and metabolic pathways, indicating an antigen-experienced, activated status of these graft-derived T cells (Fig. 2E–G). Taken together, these results suggest that persistent donor T cells receive ongoing TCR-mediated signals within the host immune environment and are potentially alloreactive. However, homeostatic proliferation and responses to viral reactivation may also contribute to activation of persisting donor T cells.

### Some host-derived T cells survive conditioning and alloHSCT treatment

**Fig. 3:**
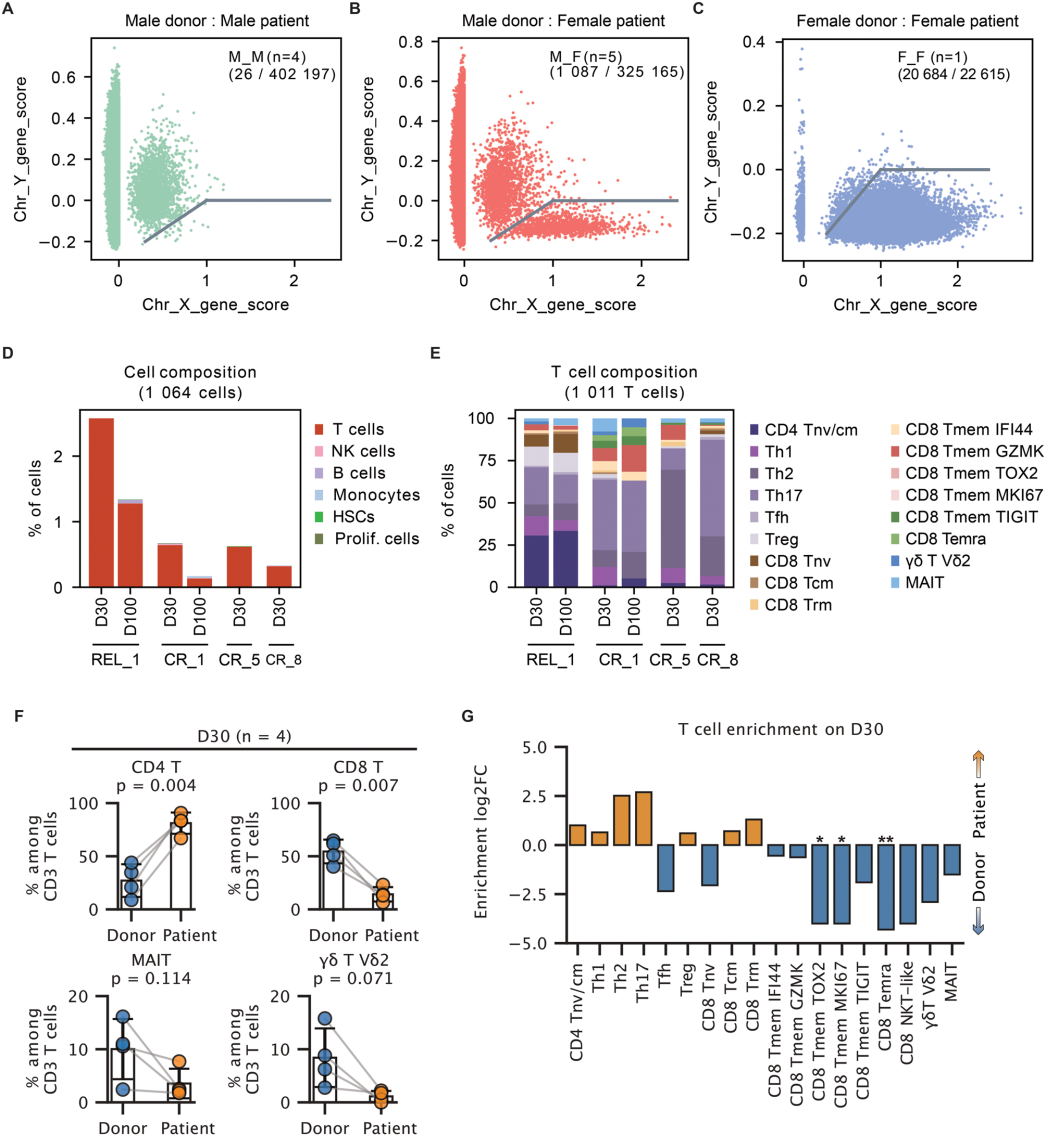
Characteristics of residual host cells in peripheral blood. (**A-C**) Chromosome X and chromosome Y related gene scores of all cells (doublets excluded) from 4 male-to-male transplantation (**A**), 5 female-to-male transplantation (**B**) patients and 1 female-to-female transplantation (**C**) patients. The female cells are gated. (**D**) Cell composition of major immune cell types from female patients gated in (**B**). Total cell counts per type are indicated. (**E**) T cell subtype composition from female patients gated in (**B**). Total cell counts per subtype are indicated. (**F**) Comparison of T cell population between donor- and patient-derived cells (patient-derived cells were gated in **B**, **D**, **E**). P-values were calculated using a two-sided paired Student’s t-test. (**G**) Differential abundance of T cell subtypes between four paired donor and patient (as indicated in **F**) at Day 30. Bars represent log2 Fold changes, with colors indicating cell origin. P-values were calculated using a two-sided paired Student’s t-test. *p*< 0.05 (*); *P* < 0.01 (**)

Measurable (minimal) residual disease (MRD) is a strong indicator of the risk of relapse of AML patients after alloHSCT treatment. We thus employed the single nucleotide polymorphism-based demultiplexing algorithm-souporcell [46] to find potential host-derived MRD, however we did not detect re-appearing leukemia cells using this tool. However, we could successfully use sex-chromosome-related genes to distinguish the cell origin in female patients who received the male-derived grafts (Fig. 3A). Compared with the cells from male patients who received male grafts, we observed a distinct cell population comprising 1,087 cells characterized with high chromosome X gene score and low chromosome Y gene score (Fig. 3A–C). Notably, 98.4% (1,070 of 1,087 cells) of these cells originated from post-transplantation samples, which served as an internal control validating our method to detect residual host leukocytes (Fig. S6A). After excluding the 17 graft-derived cells as false-positive the remaining 1,064 cells were classified as residual host cells for downstream analysis (Fig. 3C; Fig. S6B). Across all samples, residual host cells predominantly consisted of T cells (Fig. 3D; Fig. S6C). Within these residual T cell populations, we observed a dominant proportion of CD4^+^ T cells relative to CD8^+^ T cells—opposite to the distribution seen in donor-derived T cells (Fig. 3E–F). In particular, terminally differentiated and proliferative CD8^+^ T cell subsets were missing within the host-derived compartment (Fig. 3G). Collectively, high-throughput scRNA-seq analysis reliably identified residual female host cells in the peripheral blood following male-to-female alloHSCT, revealing that the T cell composition of host-derived cells was distinctly different from that of engrafted donor T cells.

### *MDGA1* marks aGvHD-associated T cells

**Fig. 4:**
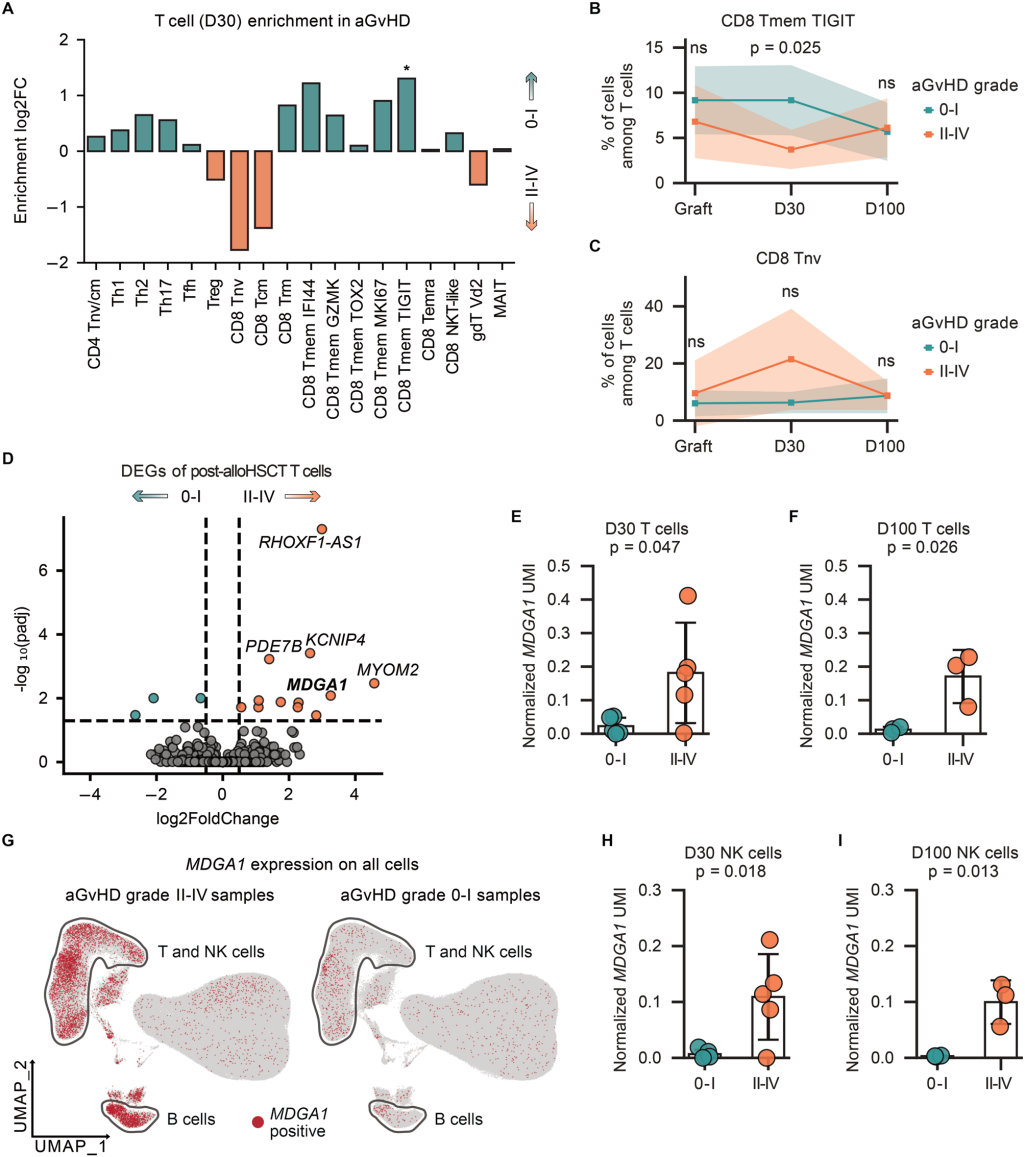
Identification of *MDGA1*^+^ T cells as a biomarker associated with aGvHD. (**A**) Differential abundance of T cell subtypes between different aGvHD outcomes at Day 30. Bars represent log2 Fold changes, with colors indicating aGvHD outcomes. P-values were calculated using a two-sided Student’s t-test. *p* < 0.05 (*). (**B-C**) Comparison of CD8 Tmem TIGIT (**B**) and CD8 Tnv (**C**) cells between different aGvHD outcomes over three timepoints. Lines are colored by aGvHD outcomes. P-values were calculated using a two-sided Student’s t-test. Not significant (ns). (**D**) Differentially expressed genes of post-alloHSCT T cells between different aGvHD outcomes. Dots are colored by aGvHD outcomes. (**E-F**) Comparison of normalized *MDGA1* UMIs of T cells between different aGvHD outcome at D30 (**E**) and D100 (**F**). Each dot represents one patient. P-values were calculated using a two-sided Student’s t-test. (**G**) Distribution of *MDGA1*-positive cells on the UMPA of all cells from aGvHD grade II-IV (left) and 0-I (right) samples. T and NK cells, B cells are indicated. (**H-I**) Comparison of normalized *MDGA1* UMIs of NK cells between different aGvhd outcome at D30 (**H**) and D100 (**I**). Each dot represents one patient. P-values were calculated using a two-sided Student’s t-test. P-values were calculated using a two-sided Student’s t-test

AlloHSCT‑associated aGvHD is a systemic inflammatory syndrome triggered by tissue damage from the conditioning regimen and subsequently sustained by donor-derived T cells [47]. First, we systematically compared the abundance of distinct T cell subset between two aGvHD outcomes and observed a relative decrease inTreg cells, naive CD8^+^ cells, and CD8^+^ Tmem TIGIT^+^ populations in aGvHD patients at D30 (Fig. 4A–B). The latter population is characterized by upregulation of exhaustion markers *TIGIT* and *TOX* with reduced cytotoxic gene expression like *GZMB*, *PRF1*, and *CX3CR1* while maintaining the expression of memory markers including *TCF7*, and *SELL* (Fig. S2A). These CD8^+^ TIGIT^+^ Tmem cells have been described as an exhausted yet persistent subset that may play a regulatory role during chronic antigen exposure. Their relative depletion may contribute to unchecked immune activation in aGvHD, although this remains speculative and warrants functional validation. Meanwhile, we observed a larger population of naïve CD8^+^ Tnv in aGvHD patients at D30, although the difference was not statistically significant (Fig. 4C). Next, we investigated whether in contrast to the subtle differences in cell population frequencies between different outcomes, aGvHD-driven systemic inflammation may exert a broader impact on the entire T cell compartment. To further identify potential cellular biomarkers, we directly compared the differentially expressed genes (DEG) of T cells and identified several upregulated genes in the aGvHD group, such as *PDE7B*, *MYOM2*, *KCNIP4*, and *MDGA1* (Fig. 4D). Of those genes, only *MDGA1* showed consistent and significant upregulation in all post-alloHSCT samples from aGvHD patients without individual dominance (Fig. 4E–F). MDGA1 encodes a glycosylphosphatidylinositol (GPI)-anchored cell surface glycoprotein, of which the function of in the immune system is currently unknown. We found that MDGA1 was exclusively expressed by lymphocytes including T cells and B cells, but barely by myeloid cells (Fig. 4G). Notably, *MDGA1* expression on NK cells was also significantly upregulated in aGvHD patients (Fig. 4H–I). Together, these data suggest that *MDGA1* expression by lymphocytes may be a candidate novel biomarker of aGvHD-associated inflammation. Nevertheless, although none of the patients included in this cohort experienced significant viral reactivation of CMV, EBV, HHV6, HSV or bacterial infection within the observation period, it is conceivable that responses to other pathogens may contribute to induce MDGA1 expression.

### *MDGA1* expression is induced by proinflammatory cytokines

**Fig. 5:**
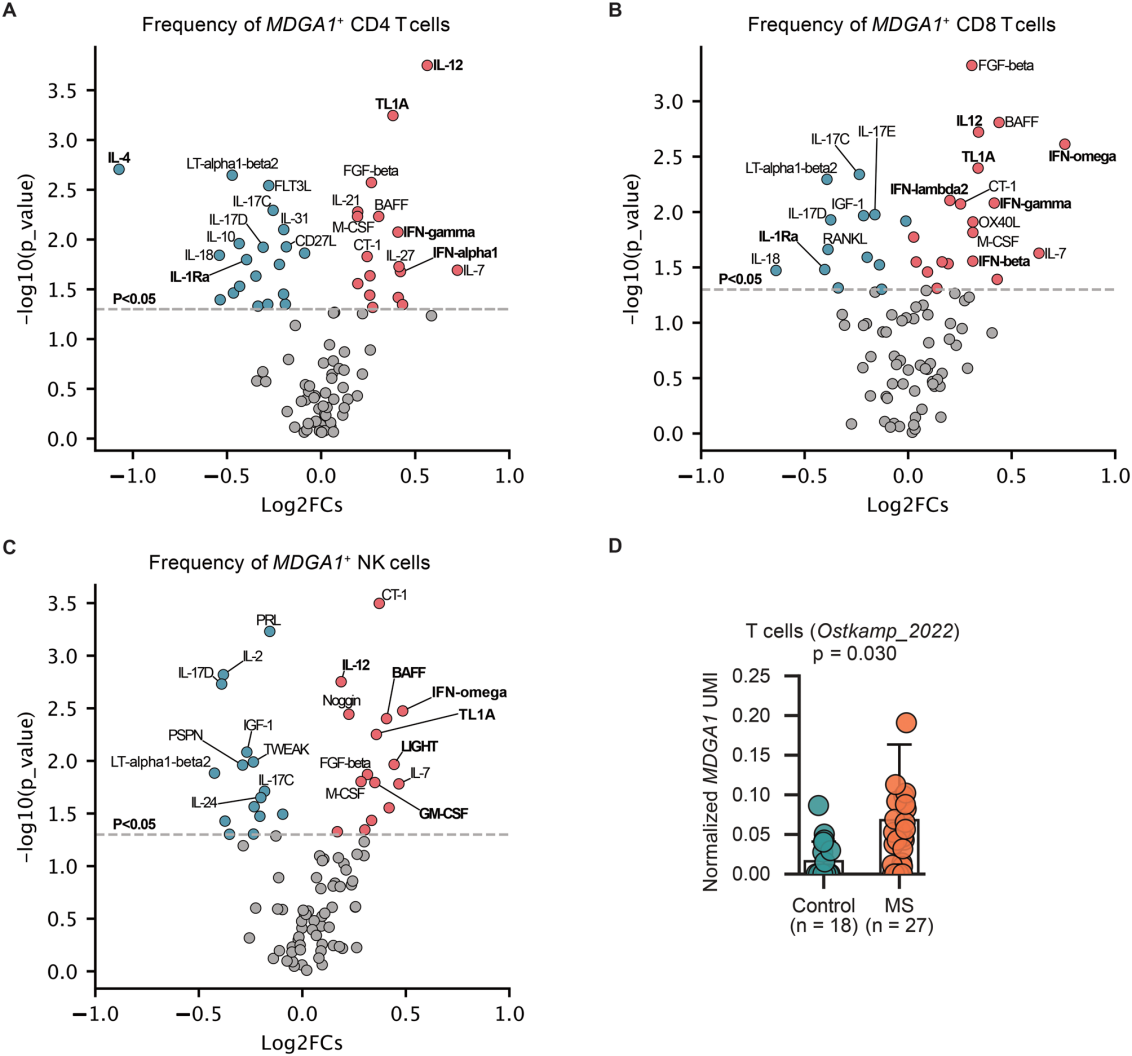
Screen cytokines for regulating *MDGA1* expression. (**A-C**) Volcano plot showing significant changes in *MDGA1*-positive CD4^+^ T cells (**A**), CD8^+^ T cells (**B**), and NK cells (**C**) of samples treated with indicated cytokines compared with PBS-treated controls. The gray dashed line represents a significance threshold of *p* = 0.05. P-values were calculated using a two-sided paired Student’s t-test. (**D**) Comparison of normalized *MDGA1* UMIs of T cells in PBMC between healthy donors and patients with multiple sclerosis. Each dot represents one patient. P-values were calculated using a two-sided Student’s t-test

Next, we hypothesized that *MDGA1* expression at the onset of aGvHD would be regulated by inflammatory cytokines. To validate this, we reanalyzed a publicly available scRNA-seq dataset which contains 10 million PBMCs from twelve healthy female and male donors treated with 90 different cytokines for 24 hours [42]. By systematically comparing the frequencies of *MDGA1* positive T in cytokine treated samples with PBS treated samples, we observed that treatment with pro-inflammatory cytokines such as IL-12, interferons, and TL1A consistently induced the expression of *MDGA1* on CD4^+^, CD8^+^, and NK cells. Conversely, anti-inflammatory cytokines such as IL-4, IL-1Ra, IL-10, and TGF-beta1 significantly downregulated the expression of *MDGA1* expression on T cells (Fig. 5A–B, Fig. S7A-B). Furthermore, we sought to validate the upregulation of *MDGA1* expression in T cells of aGvHD patients in an exploratory in vitro assay. Quantitative real time PCR analysis of sorted CD3^+^ cells showed indeed higher expression in T cells derived from alloHSCT‑associated aGvHD (Fig. S8). To additionally validate *MDGA1* expression as a biomarker for other inflammatory diseases, we analyzed a large publicly available single-cell dataset of PBMCs from multiple sclerosis (MS) patients [41]. Consistently with our data, we noticed an increased expression of *MDGA1* on T cells in MS as compared to control samples (Fig. 5D). Together, these results support the hypothesis that inflammatory cytokines induce *MDGA1* expression on peripheral lymphocytes during aGvHD, with some patients exhibiting early upregulation prior to clinical onset, suggesting—though not definitively proving—a potential role in the preclinical phase of GvHD.

### *ADGRG1* marks GvL-associated cytotoxic T cells

**Fig. 6:**
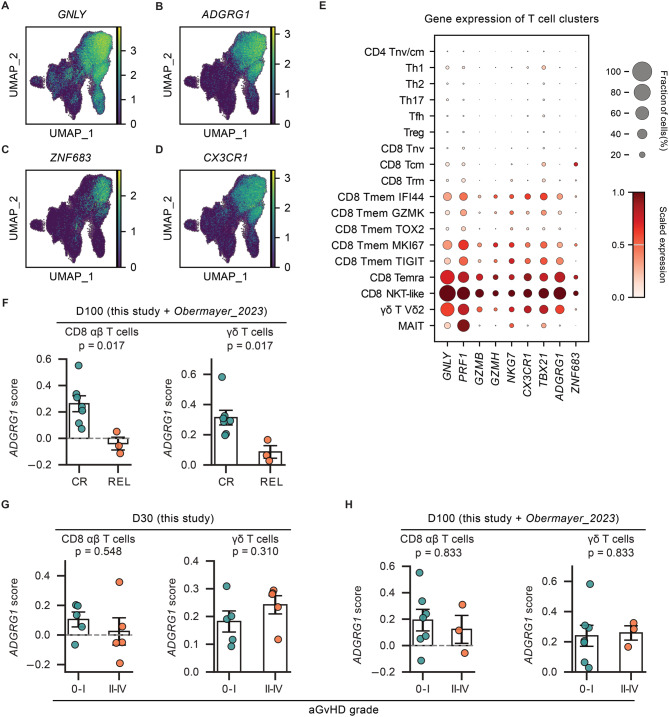
Characterize *ADGRG1* expression on peripheral T cells. (**A-D**) Normalized gene expression on the T cell UMPA. (**E**) Dot plot showing the cytotoxicity-related gene expression on T cell subtypes. (**F**) Comparison of *ADGRG1* feature on CD8^+^ T and γδ T cells at D100 between two remission status. P-values were calculated using a two-sided Mann-Whitney U test. (**G-H**) Comparison of *ADGRG1* feature on CD8^+^ T and γδ T cells between different aGvhd outcomes at D30 (**G**) and D100 (**H**). P-values were calculated using a two-sided Mann-Whitney U test

Given recent evidences identifying *ADGRG1* (GPR56) and *ZNF683* (HOBIT) as biomarkers of alloreactive cytotoxic T lymphocytes mediating GvL in the bone marrow [19, 21], we investigated whether these markers were similarly informative in peripheral blood. We firstly compared the expression patterns of *ADGRG1* and *ZNF683* with those of classical cytotoxic genes in peripheral blood CD3^+^ T cells and observed that *ADGRG1* consistently marked cytotoxic αβ T and γδ T cells (Fig. 6A–E, Fig. S9A-E). These findings indicate that *ADGRG1* functions as a shared marker for both cytotoxic αβ T and γδ T cells in the peripheral blood of AML patients.

To explore if *ADGRG1* expression on the peripheral T cells is associated with the remission status, we calculated the gene module score of *ADGRG1* using the top 20 upregulated genes of *ADGRG1*^+^ T cells identified in the bone marrow of AML patients. [21] To overcome the limited number of relapse patients in our cohort (*n* = 2), we incorporated a publicly available scRNA-seq dataset analyzing the graft and PBMCs at day 100 after alloHSCT. [43] That study by Obermayer et al. included single cell analysis of five alloHSCT patients treated for heterogenous underlying leukemia diseases, see supplementary table S3. Combined exploratory validation analysis suggested that the *ADGRG1* gene feature of CD8^+^ αβ T and γδ T was significantly reduced in patients who relapsed later (Fig. 6F). However, the *ADGRG1* gene feature of CD8^+^ αβ T and γδ T are not associated with aGvHD (Fig. 6G–H). These data are consistent with a recent report on bone marrow T cell signatures [21] and collectively suggest that *ADGRG1* expression of circulating cytotoxic T cells also is a potential biomarker of effective GvL responses in AML patients following alloHSCT.

## Discussion

AlloHSCT remains the standard of care for AML patients, but the lack of robust biomarkers for rapid diagnosis of complications, including GvHD, and risk stratification limits timely intervention and affects the optimization of clinical outcomes. In this study, we used a cutting-edge ultra-high throughput scRNA-seq strategy to profile the reconstituted immune system at single-cell resolution in AML patients. This allowed us to dynamically and longitudinally track the entire engrafted immune cell landscape and explore the association of immune cell phenotypes with clinical outcomes, thereby enabling transcriptome-wide identification of novel biomarkers of alloHSCT-associated GvL and aGvHD.

The advancement of combinatorial scRNA-seq enabled us to exponentially increase the data size without introducing technical batch effects, which is one of biggest challenges for scRNA-seq analysis. [48–52] We profiled the transcriptome of over 777,000 cells from 25 samples of ten patients in one single experiment. Based on these high-quality data, we confidently identified rare residual host-derived T cells using sex-chromosome-related genes. These host-derived T cells exhibited a markedly different phenotype compared to engrafted donor T cells with a lack of terminal differentiated CD8 T cells, which may underlie their impaired anti-leukemia activity.

Using the T cell receptor sequences as cell-intrinsic molecular identifiers, we observed multiple persistent αβ T and γδ T cell clones post-alloHSCT, which displayed alloreactivity characterized by clonal expansion and increased activity of the cell cycle pathway. Engrafted B cells were not found post-alloHSCT, which is consistent with a previous study that chemotherapy during the conditioning regimen resulted in an almost complete depletion of B cells. [53] Intriguingly, we did not observe persisting lymphocytes in REL patients, possibly leading to relapse due to lack of effective GvL. [19]

Recent studies have demonstrated that cytotoxic αβ T cells and γδ T cells are beneficial for alloHSCT treatment in AML patients. [14, 15, 19, 21] However, a universal biomarker of GvL-associated cytotoxic lymphocytes remains undefined. Recent studies found that the remission status of AML patients after alloHSCT was associated with an *ADGRG1*^+^ signature of CD8^+^ T cells in the bone marrow. Here, we identified *ADGRG1* (also known as GPR56) as a robust biomarker consistently expressed across peripheral cytotoxic αβ T, and γδ T cells, with cell abundance significantly correlated to AML remission status. Although our patient cohort contained only two REL patients, which clearly poses limitations on drawing general conclusions, this advocates for further exploring *ADGRG1* expression by all subsets of cytotoxic T cells in the peripheral blood as a general and easily accessible biomarker for monitoring and predicting AML remission after alloHSCT. [20]

The discovery of MDGA1 as a potential novel biomarker for inflammation, and in particular for GvHD in AML patients after alloHSCT was only possible due profiling of significantly larger numbers of cells and samples without introducing additional technical batch effects. Additionally, transcript detection sensitivity has been dramatically improved through the inclusion of random primers during reverse transcription. [48] Prior studies of aGvHD primarily focused on the known molecules associated with immune cell functions, such as the chemokine receptor CXCR3 on T cells and integrin CD11b on monocytes [22], while MDGA1 has not yet been associated with inflammatory lymphocytes and may thus represent a truly novel biomarker. Also, MDGA1 expression at the cell surface would make it tangible for detection by flow cytometry and thus qualify as an easily accessible biomarker. However, anti MDGA1 monoclonal antibodies are not commercially available, which also precludes protein-level validation in this study. Moreover, patients included in the current cohort were heterogenous, not all patients developed aGvHD on D30. While we here focused on high resolution reconstruction of the dynamics of post-transplantation immune cell reconstitution, including the discovery of the novel putative aGvHD biomarker MDGA1, the small and heterogeneous patient cohort remains a major limitation of this study. Therefore, these findings should be interpreted as hypothesis-generating and robustness of *MDGA1* as an aGvHD biomarker and its precise role in inflammatory responses still require exploration in larger patient cohorts and prospective sampling at the onset and resolution of aGvHD. Nevertheless, we also observed upregulation of MDGA1 on peripheral T cells from patients suffering from the inflammatory autoimmune disease MS, which supports the hypothesis that MDGA1 expression by lymphocytes is not specific to aGvHD in alloHSCT patients, but may serve as a broader indicator of inflammation.

## Conclusions

We applied ultra-high-throughput (mega-scale) combinatorial barcoding-based scRNA-seq [48] to profile the dynamics of immune cell reconstitution from graft through two follow-up peripheral blood samples of ten AML patients who received alloHSCT treatment. We demonstrated that engrafted αβTCR^+^ and γδTCR^+^ effector T cells persisted in the host and exhibited enhanced activity, particularly through TCR signaling pathways. Importantly, we identified *MDGA1* and *ADGRG1* as potential complementary markers for functionally distinct T cell subsets associated with aGvHD and GvL, respectively. Our study provides a comprehensive, longitudinal characterization of immune responses related to alloHSCT in AML patients. Therefore, we suggest that these cell surface receptors serve as novel biomarkers that could facilitate the rapid diagnosis of alloHSCT outcomes and delineate the T cell subsets associated with them. This would enable timely interventions to optimize treatment efficacy and patient outcomes.

## Electronic supplementary material

Below is the link to the electronic supplementary material.

**Supplementary Material 1: Supplementary Figures S1–S9**

**Supplementary Material 2: Supplementary Figure Legends**

**Supplementary Material 3: Supplementary Table 1**

## Data Availability

Single-cell sequencing raw data have been deposited in the Gene Expression Omnibus (GEO, RRID:SCR_005012) under accession number GSE295359. Processed single-cell AnnData objects are available at Zenodo (10.5281/zenodo.15264188). Code for data processing and analysis is deposited on GitHub (https://github.com/isihh-uke/aml-sc.git) (RRID:SCR_002630). All data and code for reproducibility will be made publicly available upon manuscript acceptance.

## Abbreviations

scRNAseq: Single-cell RNA sequencing
aGvHD: Acute graft-versus-host disease
GvL: Graft-versus-leukemia
AML: Acute myeloid leukemia
alloHSCT: Allogeneic hematopoietic stem cell transplantation
NK cells: Natural killer cells
MHC: Major histocompatibility complex
REL: Relapse
PBMC: Peripheral Blood Mononuclear Cell
D30: Day 30
D100: Day 100
DMSO: Dimethylsulfoxid
DAPI: 4’,6-diamidino-2-phenylindole
Hg38: Homo sapiens (human) genome assembly GRCh38 (hg38)
UMAP: Uniform Manifold Approximation and Projection
HVGs: Highly variable genes
PAGA: Partition-based graph abstraction
IMGT: The international immunogenetics information system
CDR3: Complementarity-determining region 3
TCR: T cell receptor
BCR: B cell receptor
IGH: Immunoglobulin heavy chain
DEA: Differential expression analysis
ORA: Over Representation Analysis
MSigDB: The Molecular Signatures Database
MS: Multiple sclerosis
log₂FC: Log₂ fold change
DCs: Dendritic cells
MRD: Measurable (minimal) residual disease
