# Supplementary material for: Mega-scale single-cell profiling reveals novel biomarkers associated with acute GvHD after allogeneic hematopoietic stem cell transplantation
Source: Biomark Res. 2025 Dec 1;13:155. doi: 10.1186/s40364-025-00868-x (PMC12670839; doi:10.1186/s40364-025-00868-x)

Supplementary Fig. S1

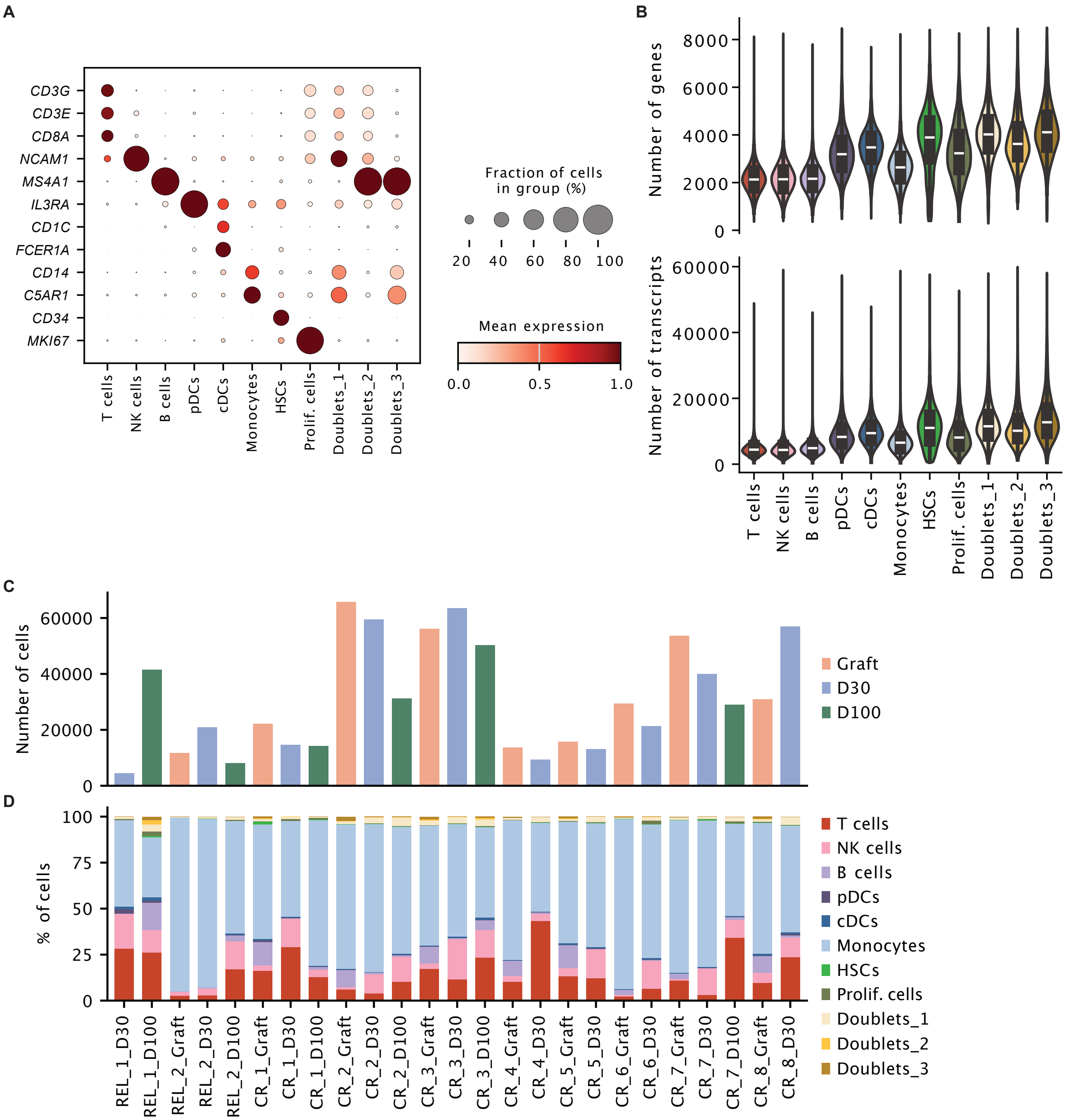

Supplementary Fig. S2

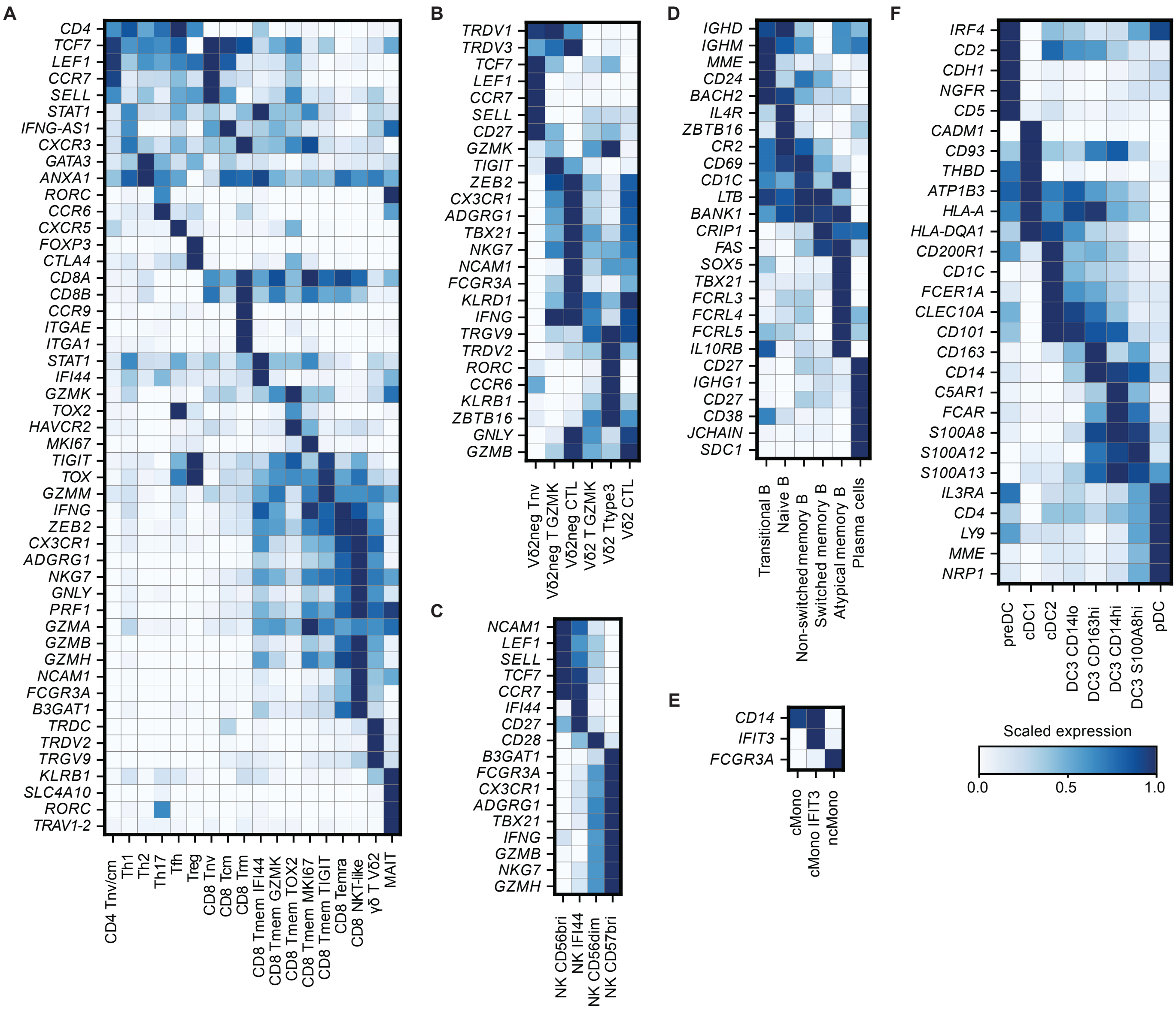

Supplementary Fig. S3

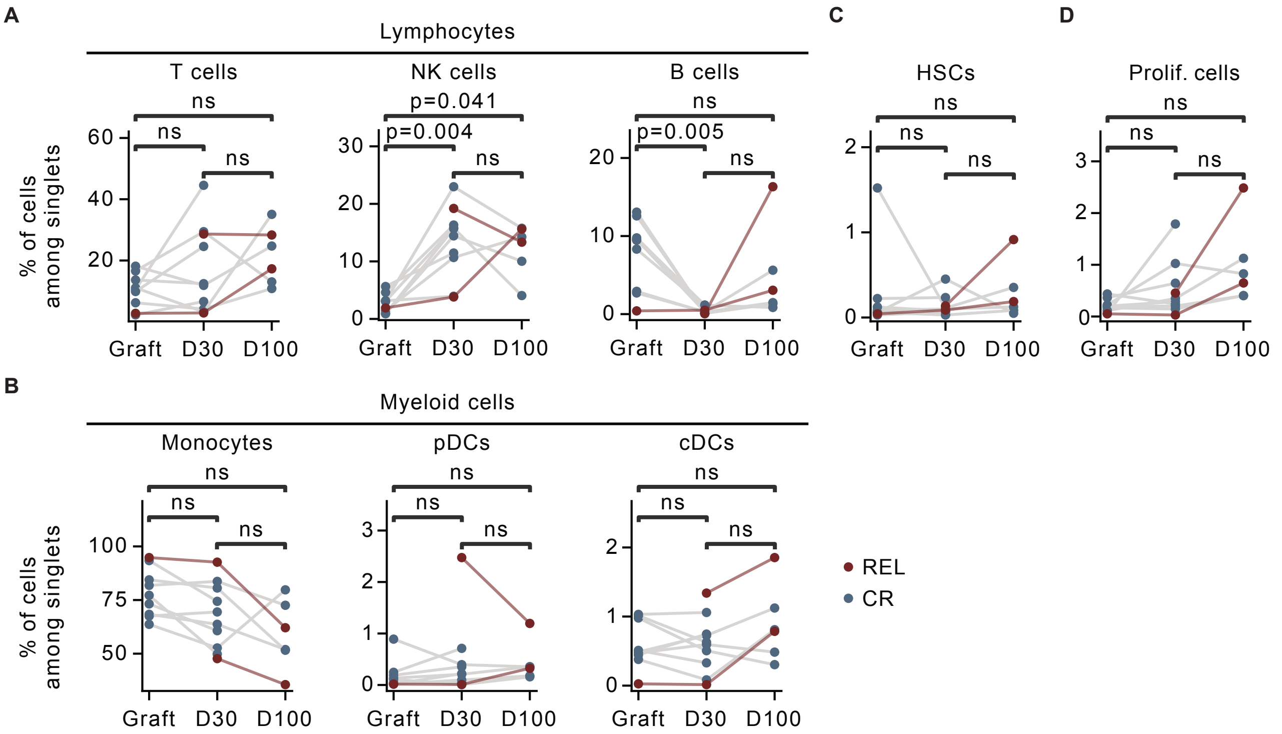

Supplementary Fig. S4

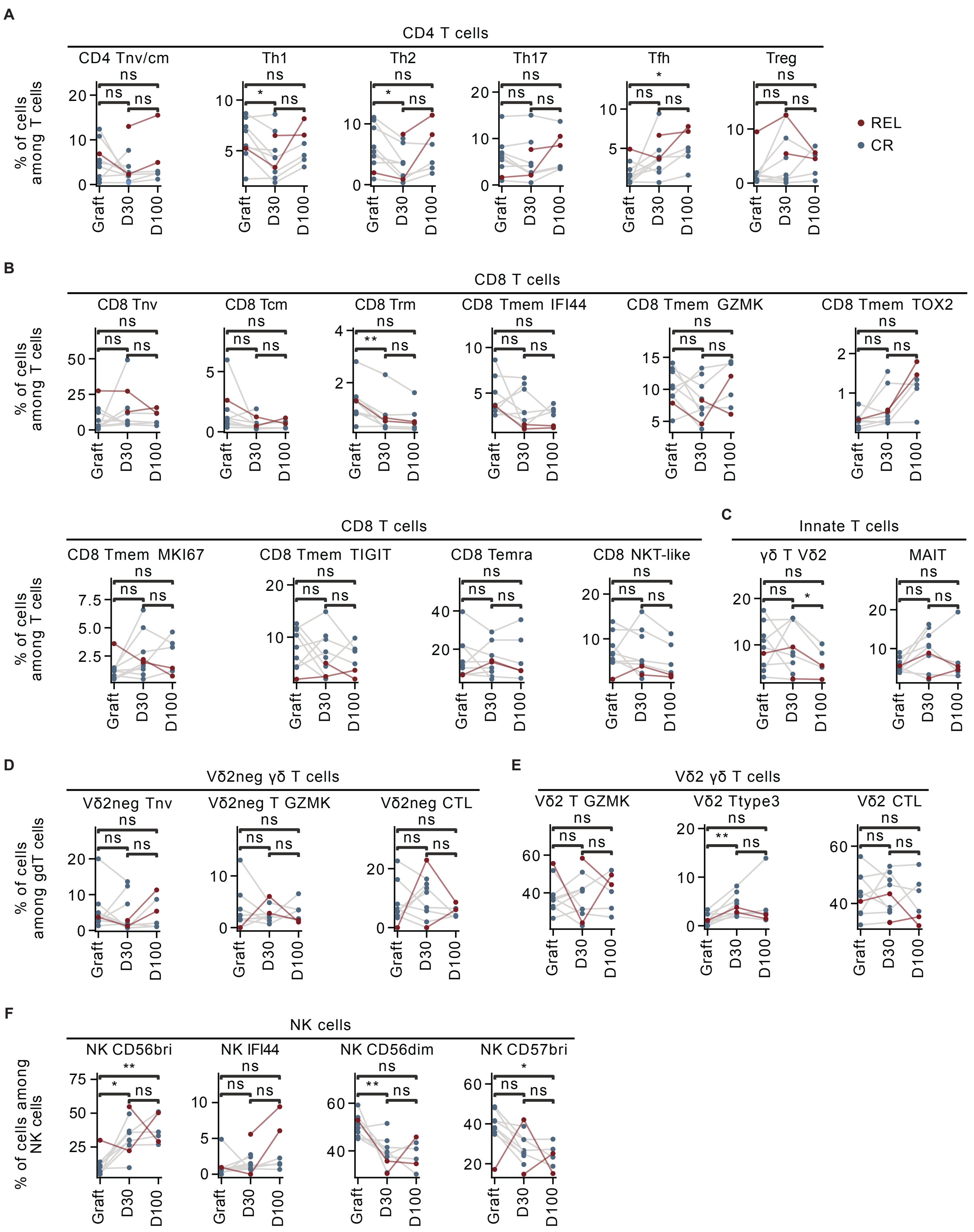

Supplementary Fig. S4

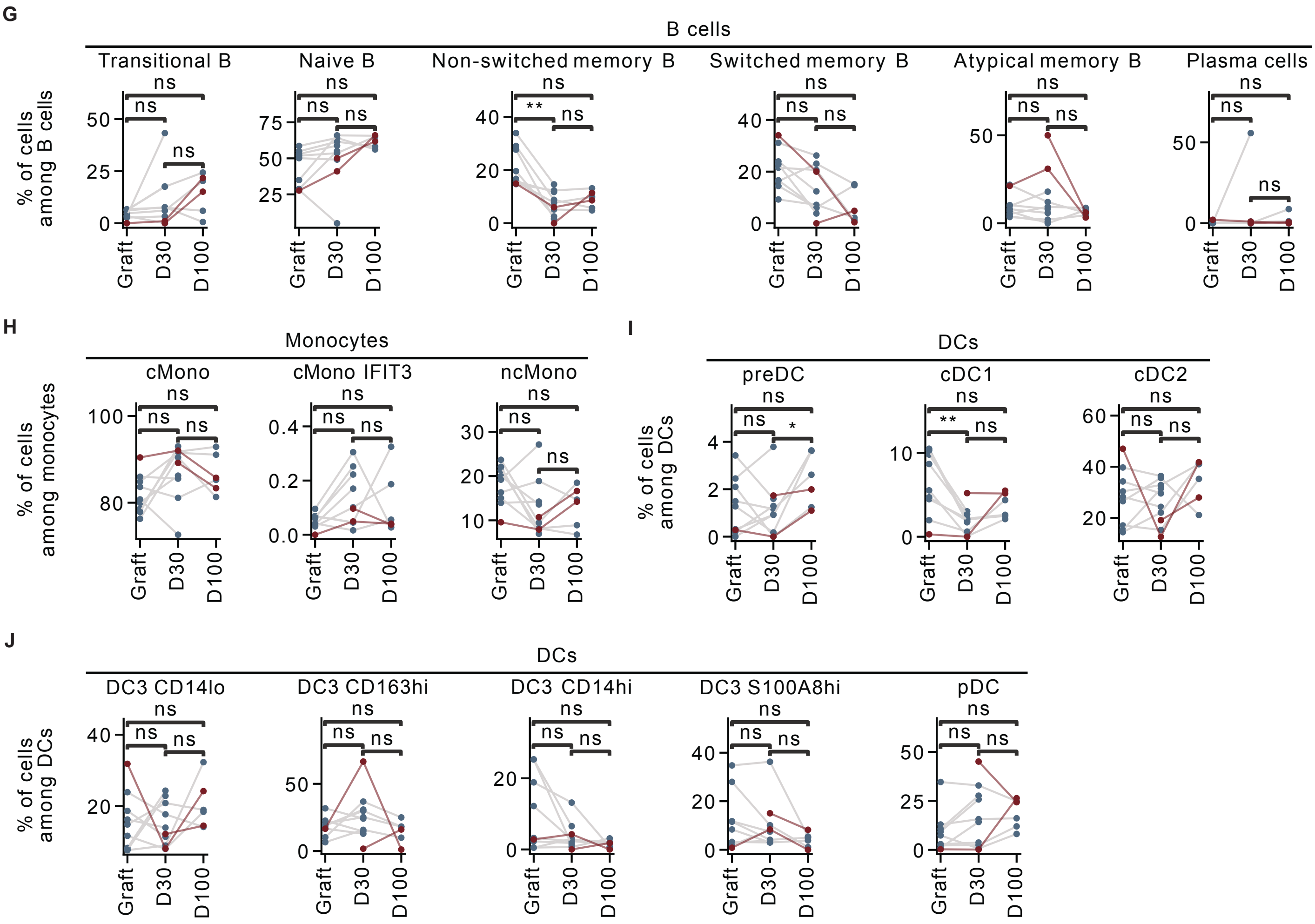

Supplementary Fig. S5

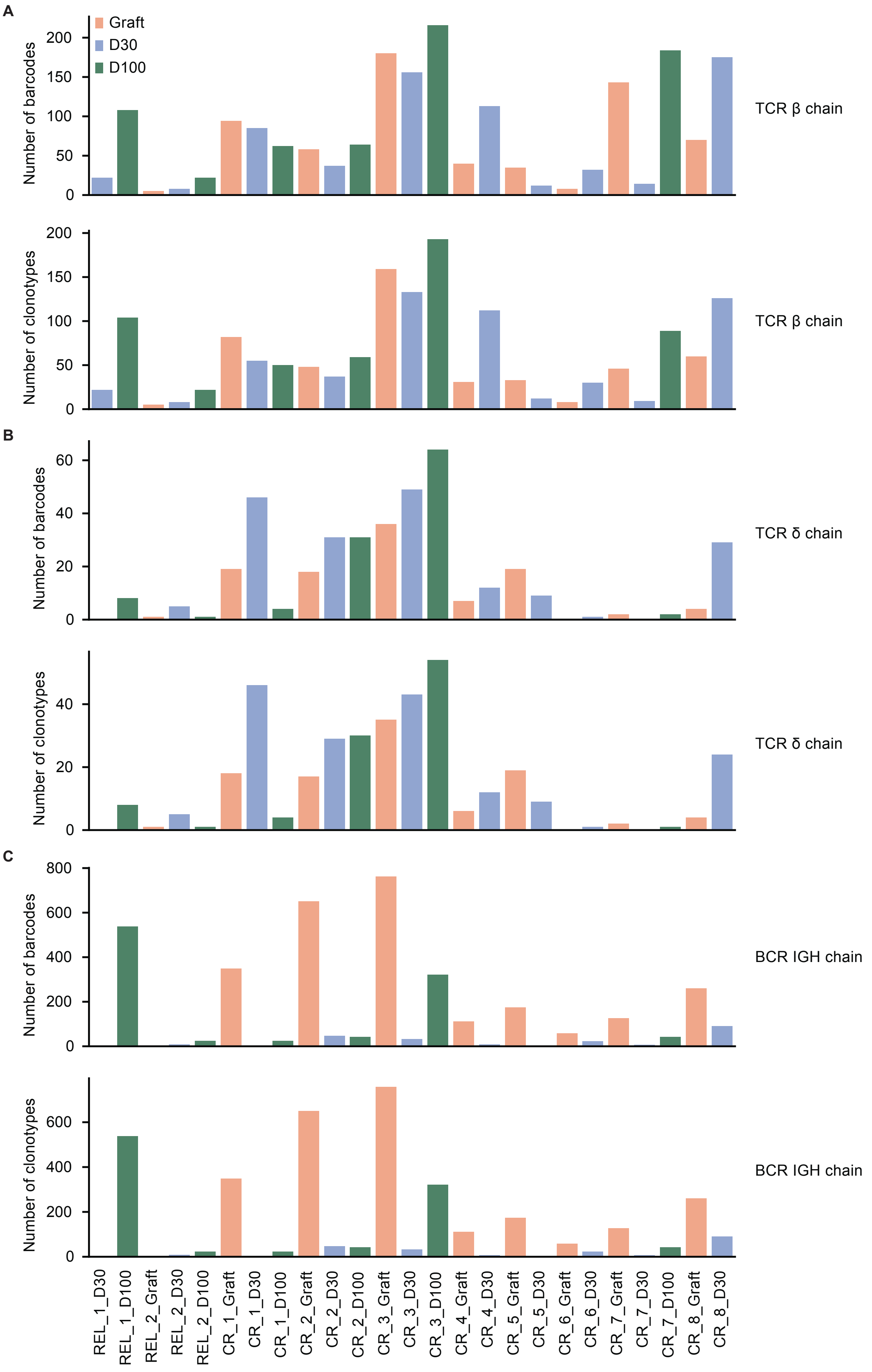

Supplementary Fig. S5

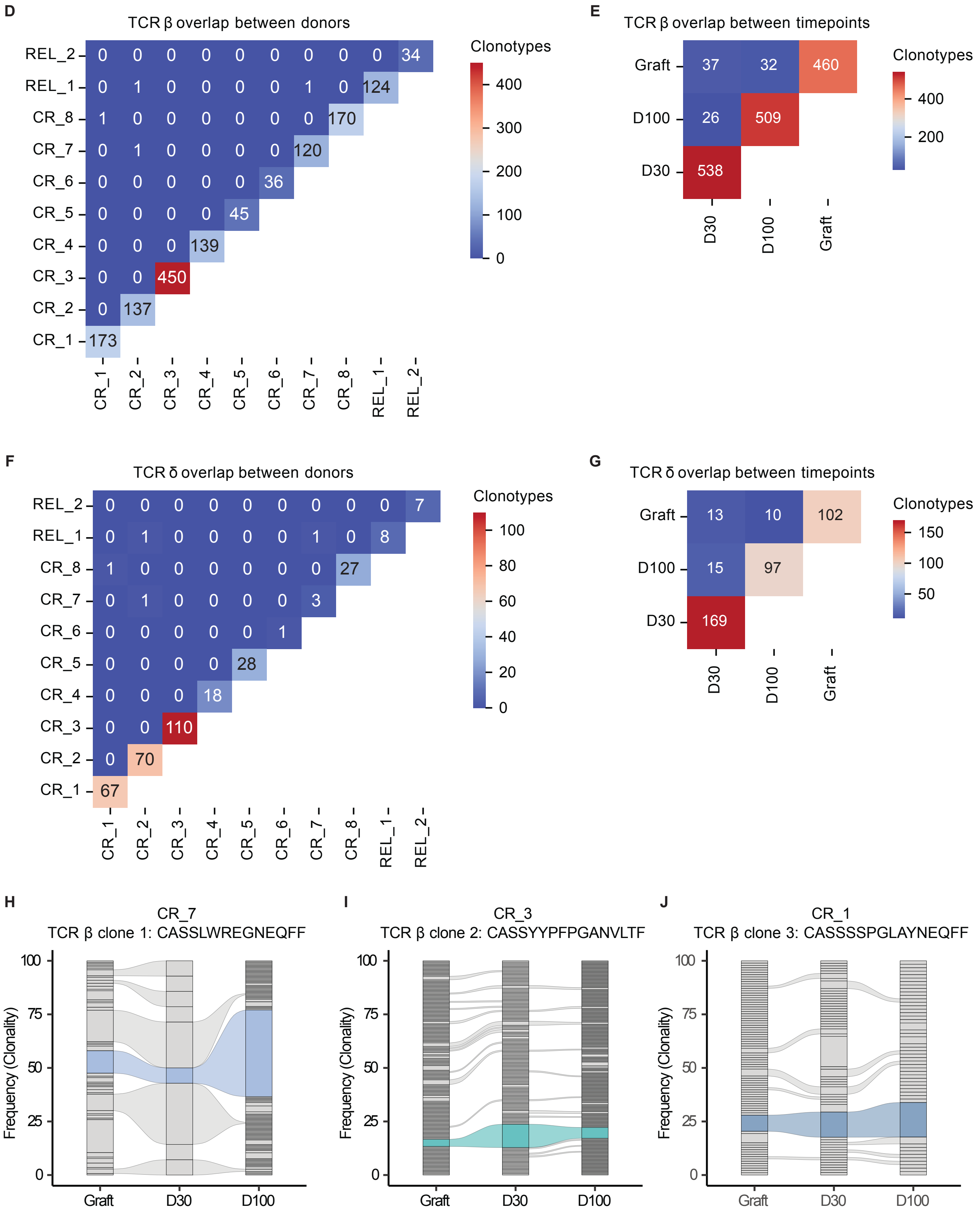

Supplementary Fig. S6

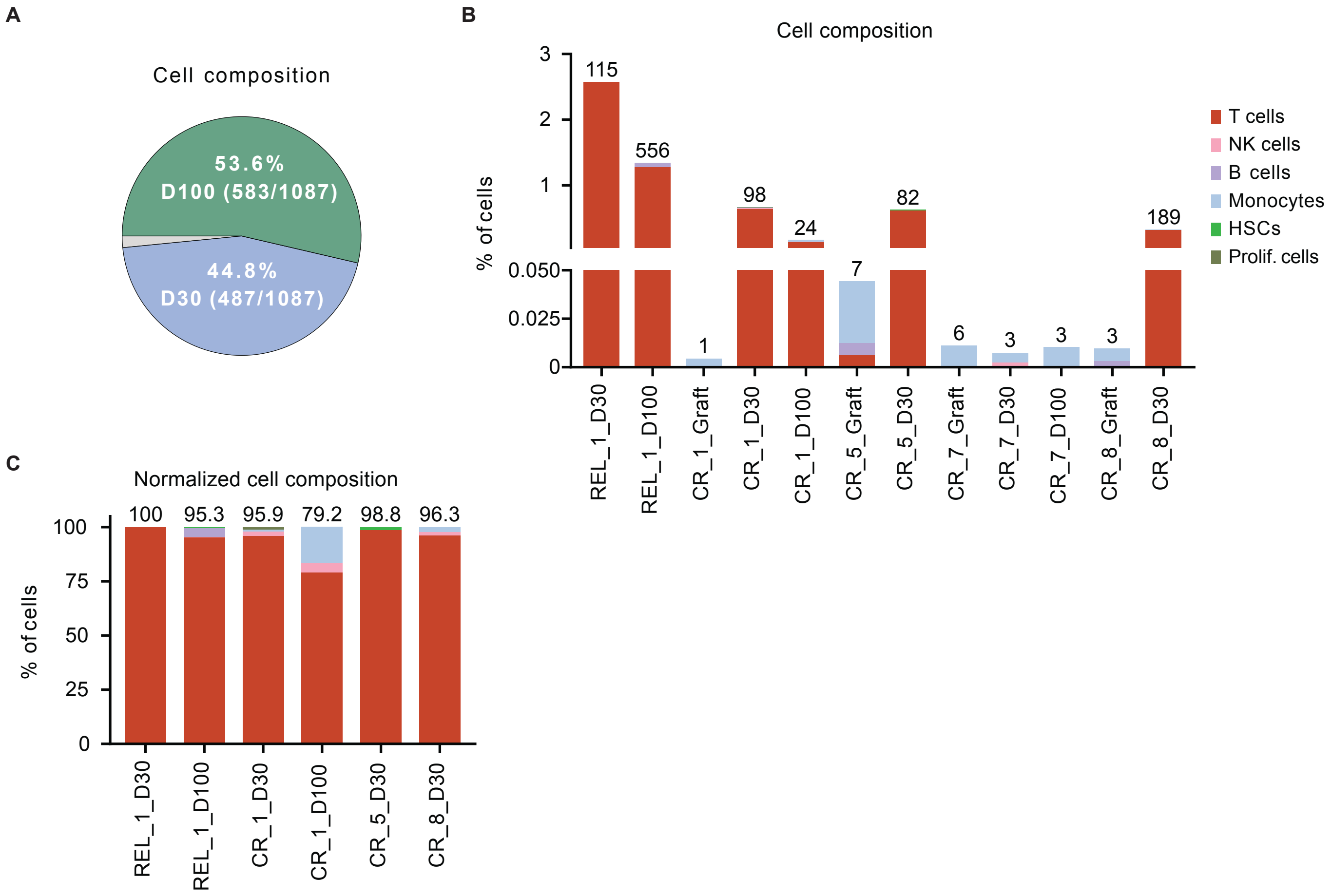

Supplementary Fig. S7

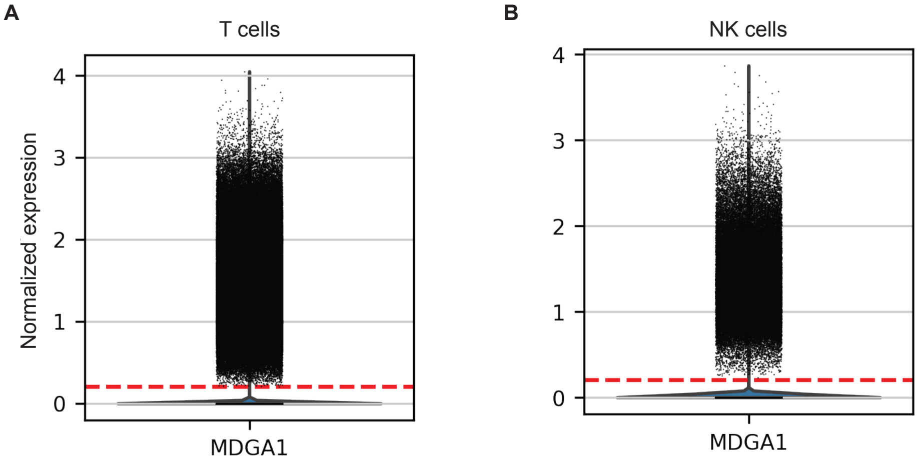

Supplementary Fig. S8

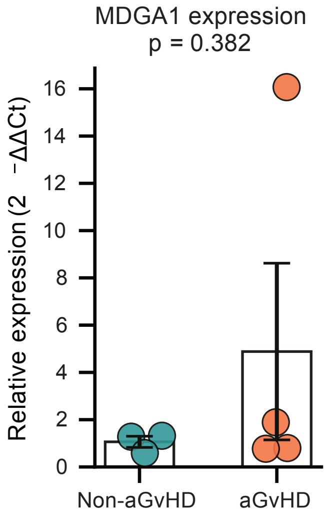

Supplementary Fig. S9

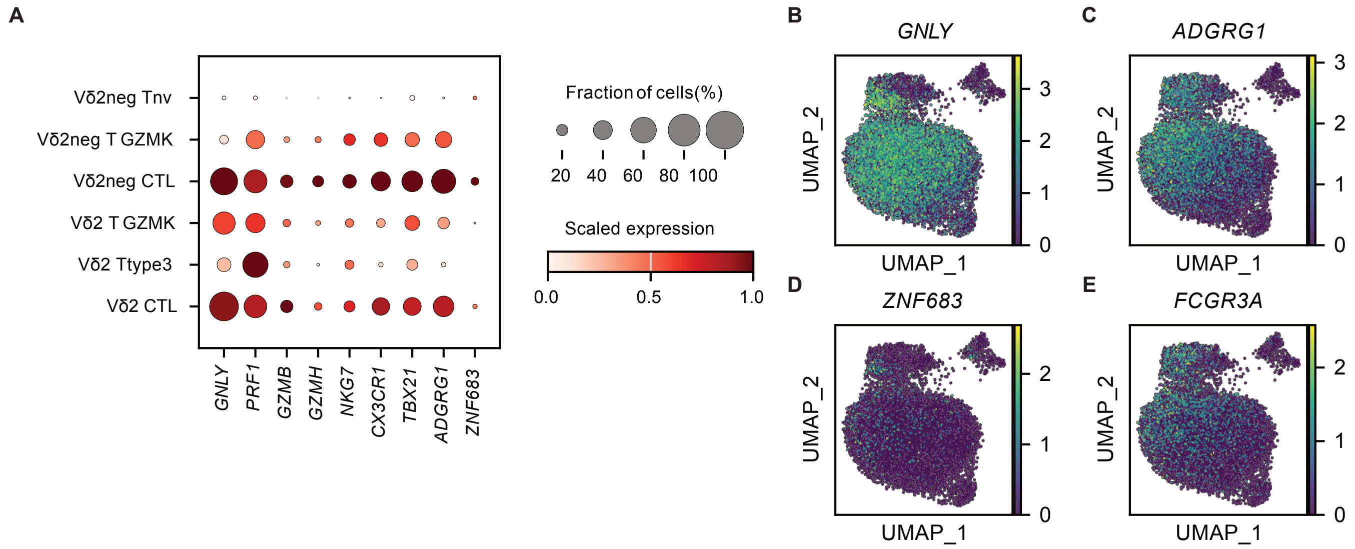

Supplement: Supplementary file 1 — Supplementary Material 1: Supplementary Figures S1–S9 [file 40364_2025_868_MOESM1_ESM.pdf]
