# Supplementary material for: Mega-scale single-cell profiling reveals novel biomarkers associated with acute GvHD after allogeneic hematopoietic stem cell transplantation
Source: Biomark Res. 2025 Dec 1;13:155. doi: 10.1186/s40364-025-00868-x (PMC12670839; doi:10.1186/s40364-025-00868-x)
Supplement: Supplementary file 2 — Supplementary Material 2: Supplementary Figure Legends [file 40364_2025_868_MOESM2_ESM.docx]

**Supplementary figure legends**

**Supplementary Fig. S1 Overview of the scRNA-seq dataset**

(A) Dotplot showing the marker gene expression of major cell populations. (B) Gene number (upper) and transcripts number (lower) of major cell populations. (C-D) Cell number (upper) and cell composition (lower) of each sample.

**Supplementary Fig. S2 Marker gene expression of subpopulations**

(A-F) Matrixplot showing marker gene expression of total T cells (A), γδ T cells (B), Natural Killer cells (NK) (C), B cells (D), monocytes (E), dendritic cells (DCs) (F). Gene expression are scaled between 0 and 1.

**Supplementary Fig. S3 Comparison of cell frequency between different timepoints**

(A-D) Comparison the frequency of lymphocytes (A), myeloid cells (B), HSCs (
C), and Prolif. cells (D). Each dot represents one sample and is colored by the remission status of the patient. P-values were calculated using a two-sided paired Student’s t-test and adjusted by Bonferroni correction.

**Supplementary Fig. S4 Comparison of subtype frequency between different timepoints**

(A-J) Comparison the frequency of CD4^+^ T cells (A), CD8^+^ T cells (B), innate T cells (
C), Vδ2neg γδ T cells (D), Vδ2 γδ T cells (E), NK cells (F), B cells (G), monocytes (H), and DCs (I-J). Each dot represents one sample and is colored by the remission status of the patient. P-values were calculated using a two-sided paired Student’s t-test and adjusted by Bonferroni correction. *: p < 0.05, **: p < 0.01.

**Supplementary Fig. S5 Overview of immune repertoire**

(A-C) The number of barcodes and clonotypes of TCR β chain (A), TCR δ chain (B), and BCR IGH chain (C) of each sample. (D-E) The overlap of TCR β clonotypes between different donors (D) or between different timepoints (E). (F-G) The overlap of TCR δ clonotypes between different donors (F) or between different timepoints (G). (H-J) The overlap and clonality of indicated clones between different timepoints.

**Supplementary Fig. S6 Overview of residual host-derived cells**

(A) Pie plot showing the cell composition of timepoints. Cell number and percentage are indicated. (B-C) The frequencies (B) and normalized frequencies (C) of residual host-derived cells from indicated samples same as that in Fig. 3B. The numbers on the top of the bar are the absolute cell number of the residual host-derived cells (B) or proportion of T cells (C). Bars are colored by cell type.

**Supplementary Fig. S7 Definition of *MDGA1*^+^ T and NK cells**

(A-B) Violin plot showing the expression of *MDGA1* on T cells (A) and NK cells (B). Cell with expression above the red dashed line (y = 0.2) was defined as MDGA1^+^.

**Supplementary Fig. S8 Validation of *MDGA1* expression in T cells from patients with acute GvHD after allogeneic hematopoietic stem cell transplantation by qPCR**

Comparison of normalized *MDGA1* expression of T cells between patients with and without aGvHD. Each dot represents one patient. P-values were calculated using a two-sided Student’s t-test.

**Supplementary Fig. S9 Characterization of ADGRG1 expression on γδ T cells**

(A) Dot plot showing the cytotoxicity-related gene expression on γδ T cell subtypes.

(B-E) Normalized gene expression on the T cell UMAP.
