# Supplementary material for: Mega-scale single-cell profiling reveals novel biomarkers associated with acute GvHD after allogeneic hematopoietic stem cell transplantation
Source: Biomark Res. 2025 Dec 1;13:155. doi: 10.1186/s40364-025-00868-x (PMC12670839; doi:10.1186/s40364-025-00868-x)
Supplement: Supplementary file 3 — Supplementary Material 3: Supplementary Table 1 [file 40364_2025_868_MOESM3_ESM.docx]

**Supplementary Table S1**

| Patient | Age | Disease | Dose of CD34^+^ cells | aGvHD | Relapse |
| --- | --- | --- | --- | --- | --- |
| A | 53 | MCL | 6 Mio/kg BW | II | no |
| B | 56 | BPDCN | 5.4 Mio/kg BW | I | no |
| C | 65 | AML | 5.3 Mio/kg BW | I | no |
| D | 54 | MDS | 6.2 Mio/kg BW | 0 | yes |
| E | 43 | AML | 4.6 Mio/kg BW | I | no |

**Supplementary Table 1:** Clinical details for the pairs A-E from public dataset *Obermayer_2023*, according to reference 43. MCL: Mantle cell lymphoma; BPDCN: Blastic plasmacytoid dendritic cell neoplasm; AML: Acute myeloid leukemia; MDS: Myelodysplastic syndrome; BW: Body weight
